# The Book World of Medicine and Science

**Published:** 1894-10-13

**Authors:** 


### THE BOOK WORLD OF MEDICINE AND SCIENCE.

Quarterly Journal of the Sanitary Institute, July, 1894. (London: Edward Stanford. Price, 2s. 6d.)

This very readable number of the journal of the Sanitary Institute is largely made up of reports of six lectures delivered at the Parkes' Museum on "Meteorology in Relation to Hygiene." These lectures exhibit both the good and the bad characteristics of the popular lecturer of to-day; they are more or less superficial, their accuracy is not always unimpeachable, but at any rate they are not inordinately dull. The position of such discourses in relation to real science is fairly put by one of the lecturers, Mr. Mill, who, after describing the effect of heat upon solids, liquids, and gases, says, "This brief summary of the physical conditions of land, water, and air with respect to heat, has been generalised and simplified to the last degree, and is no more like the actual occurrences in nature than a stiffly articulated skeleton is like a living being. . . . It is presented in strictly popular language, and is consequently on the same level of accuracy as a summary of human anatomy in which it was forbidden to call the bones or muscles by their technical names."

Mr. Scott, speaking of barometrical conditions, after giving some sketchy details concerning "caisson disease" and mountain sickness, sets himself the task of describing the hygienic influence of winds. Matters, however, do not seem to be greatly advanced in this connection by the opinions of Dr. Crothers, of Connecticut, which are quoted to the effect that, "the amount of work turned out of a Connecticut factory on damp days and days of threatening storm is said to be from 10 to 20 per cent. less than when the air is clear and the sky bright, and that the arrests made by the Chicago police exactly correspond in number with the height of the thermometer."

Nor does the subject appear to be greatly elucidated by the fancies and speculations of Mr. Ruskin in his "Storm Cloud of the Nineteenth Century," which is quoted at some length; and however interesting it may be to show the acumen of Hippocrates in finding out many things before their due time, it does not help much to the proper comprehension of the subject in hand. Discursive, however, as the lecture may be, the summing up is crisp enough. "I hope, finally, that you will carry away from this lecture the idea that the main principle which governs the healthiness or unhealthiness of a wind is whether it is giving back moisture to the earth or evaporating it, and that this depends on whether it is blowing up hill or down hill. If it ascends it is chilled, and rain falls; if it descends it is warmed and draws the moisture to itself." Meteorologically, this is good; as a matter of hygiene it is open to question.

Dr. Theodore Williams speaks with authority on "Climate in Relation to Health," and probably this will be considered the most interesting paper of the series. Not that he is always able to give reasons for what he says, but at any rate, he finds it possible to show that climate—that complex sum of many conditions—does have an influence upon health, and his experience enables him, so far as the treatment of disease is concerned, to classify climates in such a way as to define approximately the kinds suitable to the various maladies which come before the physician for consideration.

Dr. Louis C. Parkes gives a careful review of the evidence given before the Royal Commission on Metropolitan Water Supply, 1893, and he shows how complex is the problem, and how, at many points, our knowledge is so intertwined with ignorance that it becomes unsafe to dogmatise. It is interesting to note that the typhoid bacillus has never yet been discovered in the water of the Thames, and yet the evidence of those who examined the river and its tributaries is conclusive as to the abundant opportunities at present afforded for the access to it of such disease germs. It is idle then to absolve any particular water supply on the ground of even many bacteriological examinations. As to the vitality of pathogenic organisms, the wide differences between the results of various investigators show how important are the conditions under which the experiments are performed, and as it is probable that the differences *inter se* between the artificial conditions of all the laboratory experiments that have ever been performed, do not even approach the great difference which separates them all from those of a free flowing river, it is obviously unsafe to apply the results of laboratory experiments to the interpretation of nature. "They are only inferentially applicable to similar microbes in a state of nature, and subjected to a natural as opposed to an artificial environment. A similar criticism is applicable to nearly all the laboratory experiments conducted for the Commission where an attempt is made to imitate in a laboratory the circumstances and operations of a flowing river."

One of the most interesting points in regard to the continued life of pathogenic organisms outside the body, is as to how they fare in the struggle for existence when in association with ordinary invisible saprophytes. There seems no doubt that they die, or are destroyed more rapidly in water containing its normal quantum of saprophytic life than in water previously sterilised, and although it has been shown by Professor Ray Lankester that the bacterium flourescens liquifaciens can be made to live peacefully side by side with the typhoid organism, this was by no means accomplished in a state of nature, and, as Professor Lankester suggests, one ought to experiment with some thirty forms of fluviatile saprophytes under conditions resembling those obtaining in the river. One may ask, however, where such conditons can be found except actually in a river. Clearly we are still in boundless ignorance of these matters, and we can agree with Dr. Parkes when he says, "It is premature to say, as the Commissioners have done in their report, that when pathogenic bacteria pass into the outer air or water they are in an unnatural medium, in which they can only maintain their existence and power of multiplication for a limited period, during which they undergo more or less rapid attenuation or loss of virulence, and become generally weakened. Future research, indeed, the most recent investigations, will probably show that there may be most important exceptions to any such rule." Bacteriology, like statistics, may be twisted many ways, either to the satisfaction of the foolish or the confounding of the wise, and does not appear as yet to have arrived at such a position as to entitle it to be taken as a safe guide in practical affairs.

The Royal Commissioners expressly stated in their report that they regarded bacteriology as still in its infancy, and they did not consider that the results obtained by experts in the science, in regard of the persistence or destructibility of pathogenic bacteria could have any such position of absolute certainty assigned to them as would dispense with the evidence of experience. With this view all who have read the bacteriological evidence placed before the Commission will agree. There is not merely the want of harmony between the results of different observers; but there has been too little insistence on the artificiality of the experimental methods pursued, and on the danger of any reasoning from such observations being applied to the circumstances and operations of nature on the large scale. There is, besides, the almost universal tendency to regard the microbes of disease as specific entities, and to take little account of those possible modifications and reversions of types about which so little is known, but that, when understood, will probably supply a key to the solution of the most intricate epidemological problems.

